# Phase-field simulation of crack growth in cortical bone microstructure: parameter identification and comparison against experiments

**DOI:** 10.1007/s10237-025-01929-8

**Published:** 2025-03-01

**Authors:** Jenny Carlsson, Olivia Karlsson, Hanna Isaksson, Anna Gustafsson

**Affiliations:** Department of Biomedical Engineering, Lund University, Box 118, 221 00 Lund, Sweden

**Keywords:** Cement line, Osteon, Fracture toughness, Crack propagation, Phase-field method

## Abstract

Computational models are commonly used to investigate how the cortical bone microstructure affects fracture resistance; recently, phase-field models have been introduced for this purpose. However, experimentally measured material parameters for the microstructural tissues are lacking. Moreover, as no validation studies have been published, it remains unclear to what extent classical phase-field methods, assuming linear-elastic, brittle fracture, accurately represent bone. In this study, we address both these shortcomings by first applying a design-of-experiments methodology to calibrate a set of material parameters for a two-dimensional phase-field finite element model of bovine osteonal microstructure. This was achieved by comparing the outcomes from simulation to data from single-edge notched bending experiments on bovine osteonal bone and subsequent imaging of the crack path. Second, we used these parameters in new bone geometries to evaluate the parameters and the predictive performance of the model. Reasonable agreement was achieved between prediction and experiments in terms of peak load, crack initiation toughness and crack path. However, the model is unable to capture the experimentally observed gradual evolution of damage, leading to a nonlinear force response before the onset of visible crack extension. Nor does it capture the similarly observed increase in toughness with increasing crack length. These limitations are inherent to all classical phase-field methods since they originate from theories of brittle fracture, and alternative formulations are discussed. This is the first study attempting to validate classical phase-field methods in simulation of cortical bone fracture, and it highlights both potential and limitations to be addressed in future work.

**Supplementary Information:**

The online version contains supplementary material available at 10.1007/s10237-025-01929-8.

## Introduction

Osteoporosis is characterised by insufficient bone strength and an increased risk of fragility fractures, but the mechanisms behind bone fragility are poorly understood. Young, healthy bone exhibits extrinsic toughening mechanisms, which prevent unstable crack growth (Launey et al. 2010; Nalla et al. 2005), but with ageing, bone becomes more brittle (Burr 2019).^1^ For example, cracks propagating in old bone primarily grow along straight trajectories, whereas cracks propagating in young bone tend to deflect in the microstructure (Chan et al. 2009). Deflection leads to longer cracks and thus relatively higher energy dissipation, and reduced stress intensity at the crack tip (Launey et al. 2010).

Understanding the role of the healthy osteonal microstructure in preventing crack growth is an important step towards understanding why these mechanisms are lost in older bone. In humans, osteons make up about 40–70% of the cortical bone tissue (Granke et al. 2016, see also Abdel-Wahab et al. 2012; Wang et al. 2020). Osteons are thought to have higher (fracture) toughness compared to that of the interstitial bone (Mullins et al. 2009), but the toughness is not by itself sufficient to deflect cracks (Gustafsson and Isaksson 2022). The cement lines surrounding osteons have been identified as an important feature for crack deflection (Chan et al 2009; Koester et al. 2008; O’Brien et al. 2005); however, the properties of the cement line are debated. Some studies report that cement lines are highly mineralised and thus should have a higher modulus compared to the surrounding tissue (Skedros et al. 2005; Cantamessa et al. 2024). Other researchers report a cement line modulus that is lower than the surrounding tissue (Burr et al. 1988; Montalbano and Feng 2011; Zhou et al. 2020). The orientation of the mineralised collagen fibrils changes at the cement line such that fibrils are parallel to the cement line rather than orthogonal to it (Raguin et al. 2021). Osteon push-out tests have revealed that the cement line shear strength is ten times lower than in the surrounding tissue (Dong et al. 2005), but no experiments exist that measure the toughness.

Our understanding of the toughness of the cement line is thus largely based on studies using computational finite element (FE) models. In such models, the strength and/or toughness of the cement line is generally assumed to be lower than that of the surrounding material (see e.g. Abdel-Wahab et al. 2012; Gustafsson et al. 2019a; Maghami et al. 2021; Mischinski and Ural 2011). Under such assumptions, models predict crack deflections similar to experimental observations. It should be noted, however, that none of these models have been validated in comparison to experiments and, thus, that the results are solely theoretical.

Most of the previous computational studies on osteonal microstructure have employed cohesive zone models (CZM) (e.g. Mischinski and Ural 2011; Wang et al. 2020), the extended finite element method (XFEM) (e.g. Abdel-Wahab et al. 2012; Budyn and Hoc 2007; Budyn et al. 2008; Gustafsson et al. 2019a; 2019b; Shin et al. 2024; Yadav et al. 2021), or a combination of the two methods (e.g. Demirtas et al. 2016; 2023). Recently, phase-field methods have been applied to models of cortical bone microstructures (Maghami et al. 2021; 2022; Josephson et al 2022). Considering how phase-field methods have become ubiquitous in computational fracture mechanics, it seems likely that continued efforts also in the bone mechanics field will increasingly use phase-field models.

However, it is advisable to first verify the validity of phase-field models in bone application, as no validation to experimental data has been presented for available phase-field models (Maghami et al. 2021; 2022; Josephson et al. 2022). The difficulty associated with experimental characterisation means that experimentally determined material parameters on the tissue level are lacking. This is largely true also for parameters calibrated using models validated against experiments, with some exceptions, for example Giner et al. (2017), using an element-deletion method,^2^ and Shin et al. (2024) and also Yadav et al. (2020), using XFEM. But even with validated models, parameters relating to toughness and strength cannot automatically be carried over from one modelling framework to another. For example, the difference between cohesive fracture (which is the case with CZM and commonly also XFEM) and brittle fracture (which is the case in classical phase-field models) shifts emphasis from strength to toughness. In cohesive methods, crack initiation is governed by a material strength. Toughness usually plays a lesser role in terms of the crack path as it only governs the subsequent traction versus separation behaviour. While different XFEM implementations are possible, they all require that the crack is inserted based on some criterion, which is typically related to some kind of strength. In classical phase-field methods, the crack path is determined by the principle of minimum potential energy, where the toughness corresponds to the energy required to create new crack surfaces. Material strength does not exist in classical phase-field methods, which are strictly brittle in their converged form (Kumar et al. 2020). However, most materials can be both brittle and ductile, depending on the geometry and the (ductile-to-brittle) transition crack length ${a}_{c}\approx {E\mathcal{G}}_{\text{c}}/(\pi {\sigma }_{c}^{2}),$ where ${\sigma }_{c}$ is the uniaxial strength of the material, $E$ is Young’s modulus and ${\mathcal{G}}_{c}$ is the critical energy release rate. If the length parameter ${\ell}$ in classical brittle phase-field methods is chosen close to the transition crack length, then the model will predict an accurate uniaxial strength. In the case of the first-order crack density functional used in this work, commonly referred to as Ambrosio-Tortorelli 1 (AT1), this means choosing ${\ell}=\sqrt{3E{\mathcal{G}}_{c}/(8{\sigma }_{c}^{2})}$ (Pham et al. 2011). But the phase-field regularisation length must also resolve the microstructure and using as a reference length the smallest feature of the bone microstructure, the ~ 5 µm wide cement line, results in a strength of around 1000 MPa, which is clearly too high. In CZM and XFEM models on the other hand, the (ductile-to-brittle) transition crack length may be arbitrarily large without compromising the resolution of the microstructure, and the relative importance of strength contra toughness increases with the transition crack length.

Considering these differences, the first objective of this paper is to calibrate a set of material parameters for a classical phase-field model by comparison to experimental data (Gustafsson et al. 2024). This is done using a design-of-experiments (DOE) approach in two steps. The second objective is to apply the material parameters obtained in the first two steps to three new simulation geometries, also from Gustafsson et al. (2024), but from different experimental specimens, to evaluate the performance of the phase-field model for predicting mechanical response and crack paths in cortical bone specimens.

## Theory

The phase-field method for fracture, developed by Francfort and Marigo (1998) and Bourdin et al. (2000), considers fracture as an energy minimisation problem. We here consider a two-dimensional body $\Omega$, containing a crack $\Gamma$. The potential energy $\Pi$ of the system can be written as the sum of the elastic energy and the surface energy due to crack growth,

1 $$\Pi ={\int }_{\Omega }\psi d\Omega +{\mathcal{G}}_{c}{\int }_{\Gamma }d\Gamma ,$$

where $\psi$ is the elastic strain energy density. The phase-field formulation of Bourdin et al. (2000) consists of rewriting the last term using a crack density function which takes as argument a damage parameter $d$. The damage parameter varies smoothly between 0 (in undamaged material) and 1 (in completely damaged material). Material damage is accompanied by local degradation of the strain energy of the undamaged material, ${\psi }_{0}$, following a degradation function of the form ${[\left(1-d\right)}^{2}+k]$, where $k$ provides a small residual stiffness for enhanced stability (Miehe et al. 2010). Throughout this work, $k={10}^{-7}$ has been used. Using the AT1 crack density functional (Ambrosio and Tortorelli 1990), the potential energy $\Pi$ of the discrete problem (1) can be approximated by the potential energy of the regularised phase-field problem ${\Pi }_{{\ell}}$,

2 $$\Pi \approx \Pi _{\ell } = \int _{\Omega } \left\{ {\underbrace {{[\left( {1 - d} \right)^{2} + k]\psi _{0} }}_{\psi } + \frac{{3{\mathcal{G}}_{c} }}{{8\ell }}\left( {d + \ell ^{2} \nabla d \cdot \nabla d} \right)} \right\}d\Omega.$$

The length scale parameter, ${\ell}$, determines the width of the regularised crack. To prevent crack growth under compression the strain energy is split into a positive and a negative part, where only the positive part is assumed to be degraded, i.e. $\psi =[{\left(1-d\right)}^{2}+k]{\psi }^{+}+{\psi }^{-}$. The so-called hydrostatic-deviatoric split of Amor et al. (2009) is used, in which

3 $$\begin{array}{c}{\psi }^{+}=\frac{\alpha }{2}{\kappa }^{\prime}\text{tr}{\left({\varvec{\varepsilon}}\right)}^{2}+\mu {{\varvec{\varepsilon}}}_{dev}:{{\varvec{\varepsilon}}}_{dev}\\ {\psi }^{-}=\frac{1-\alpha }{2}{\kappa }^{\prime}\text{tr}{\left({\varvec{\varepsilon}}\right)}^{2}\end{array}$$

where $\alpha = 1$ if $\text {tr}({\varvec{\varepsilon}}) > 0$ and 0 otherwise, ${\varvec{\varepsilon}}=0.5(\nabla {\varvec{u}}+{\nabla }^{T}{\varvec{u}})$ is the linearised strain, ${{\varvec{\varepsilon}}}_{dev}={\varvec{\varepsilon}}-0.5\text{tr}\left({\varvec{\varepsilon}}\right){\varvec{I}}$ is the strain deviator (${\varvec{I}}$ is the identity matrix), ${\kappa }^{\prime}$ is the two-dimensional plane strain bulk modulus and $\mu$ is the shear modulus. The hybrid implementation of Ambati et al. (2015a) is used. Irreversibility of crack growth is ensured by use of a history field such that $\mathcal{H}({\varvec{x}},t)=\underset{\tau \in \left[0,t\right]}{\text{max}}{\psi }^{+}({\varvec{x}},\tau )$ (Miehe et al. 2010). A threshold value of the history field prevents negative damage values which could otherwise occur with the AT1 model (Wu and Huang 2020).

Simulations were performed using commercial finite element software Abaqus/Standard^3^ with a user element (UEL) for the phase-field formulation. The UEL was developed by Martinez-Paneda et al. (2018), and the quasi-Newton solver of Kristensen and Martinez-Paneda (2020) was implemented by Gustafsson and Isaksson (2022), all details of the implementation can be found in these references.

## Method

**Fig. 1:**
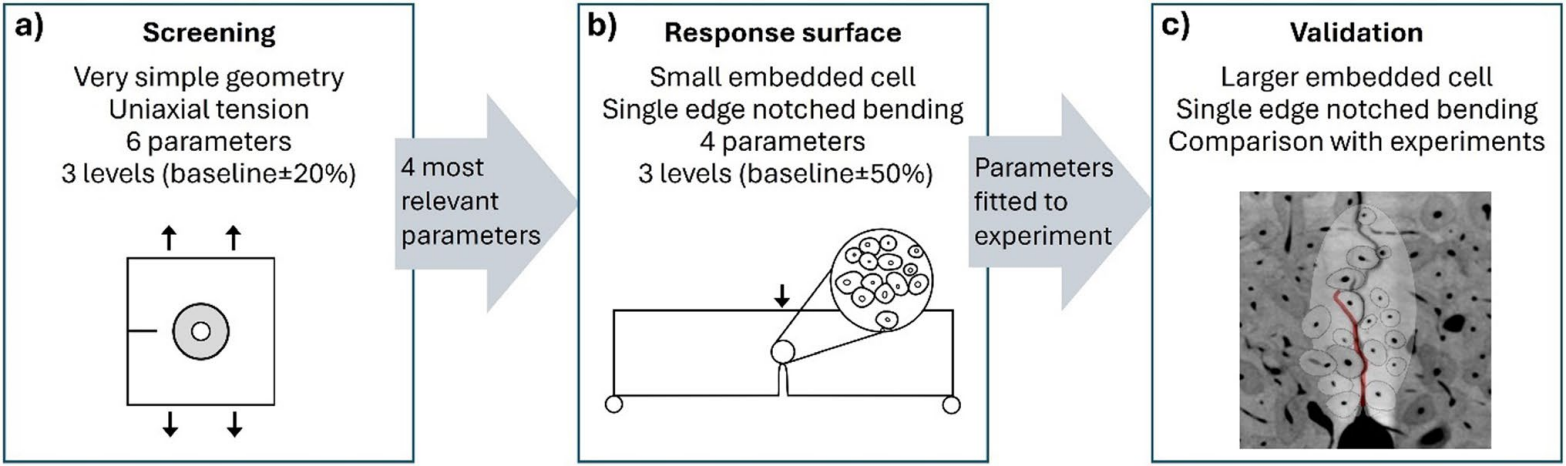
Overview of the analysis procedure. **a** Relevant parameters were identified using a simple single-osteon model. **b** A response surface analysis was performed, resulting in the set of parameters that best fits the experimental results. **c** The fitted parameters were evaluated in new geometries, based on new bone specimens, in comparison to experiments

The study involved a two-step parameter study, starting with a screening analysis considering a simple geometry to identify the most relevant parameters for the microstructural model (Fig. 1). The cortical bone microstructure was considered to be a composite consisting of three types of tissue: interstitial matrix, osteons and cement lines, each with its own material parameters. The most relevant parameters were then used in a response surface analysis, considering a single-edge notched bending (SENB) cortical bone specimen from the experimental study by Gustafsson et al. (2024). Response surfaces for peak force, crack tortuosity and toughness were generated, and a set of material parameters was obtained by comparing the response surfaces to the experimental results. In the last step, the fitted parameters were implemented in three new SENB cortical bone geometries to evaluate the performance of the model in comparison with experimental results.

### Literature study and baseline material parameters

A literature study was performed to identify reasonable ranges of values for the required material parameters, i.e. moduli and toughness for interstitial matrix, osteon and cement line tissues (Table 1). The purpose of the literature study was to identify a baseline level for the set of parameters to be varied in the parameter studies. The focus was on experimental results; numerical studies were included only to illustrate the range of values typically used in computational models. Properties obtained under wet or physiological conditions were preferred, while properties obtained for dry material were used for relative quantities only, as Faingold et al. (2014) reported similar changes in properties between wet and dry conditions for all microstructural tissue types. Based on the literature study, a set of baseline material parameters was chosen (Table 2). Due to the uncertainty of the cement line modulus, its value was chosen to be identical to that of the interstitial matrix. Poisson’s ratios were excluded from both literature and parameter studies since Gustafsson et al. (2019b), using XFEM, found that Poisson’s ratio did not contribute significantly to the results. The baseline Poisson’s ratio from Gustafsson et al. (2019b) of 0.3 was used for all tissue types.

**Table 1:** Summary of the material parameters available in the literature

Parameter	Tissue	Notation	Experimental studies	Numerical studies
Young’s modulus (GPa)	Interstitial matrix	${E}_{\text{mat}}$	13.8–15.3^a^; 13.26^b^;	14.122^1,4^; 14.6–15.4^2^; 18.5–27.1^3^; 25.8^5^; 4^6^; 14.6^7^; 19.7^8*^; 19.02^10^
	Osteon	${E}_{\text{ost}}$	0.7 ${E}_{\text{mat}}$ ^c^; 0.91 ${E}_{\text{mat}}$ ^d^; 0.85 ${E}_{\text{mat}}$ ^e^; 0.94 ${E}_{\text{mat}}$ ^f^; 0.74 ${E}_{\text{mat}}$ ^g^; 10.5^b^; 7.4–18.5^h^	13.5–14.3^2^; 16.6—25.1^3^; 9.13^4^; 22.5^5^; 4.4^6^; 13.6^7^; 16.6^8*^; 13.70^10^
	Cement line	${E}_{\text{cem}}$	${E}_{\text{cem}}$>${E}_{\text{mat}}$ ^i, j^; ${E}_{\text{cem}}$<${E}_{\text{mat}}$ ^k^; 0.7 ${E}_{\text{mat}}$ ^l^; 0.3 ${E}_{\text{mat}}$ ^f^; 0.4 ${E}_{\text{mat}}$ ^g^	0.75 ${E}_{\text{ost}}$ ^2^; 6.85^4^; 3.3^6^; 10.12^7^; 12.45^8*^; 8.61^10^
Critical energy release rate (N/mm)	Interstitial matrix	${\mathcal{G}}_{c,\text{mat}}$	0.6–0.8^m^; 0.05–0.2^n,o^; 0.05–0.13^p^; ~ 0.2^q^	1.16^3^; 0.238^4^; 0.05^5^; 0.132^7^; 0.52^8*^; 0.051^9^; 1.128^10^
	Osteon	${\mathcal{G}}_{c,\text{ost}}$	${\mathcal{G}}_{c,\text{ost}}$>${\mathcal{G}}_{c,\text{mat}}$ ^e^	0.86^4^; 0.05^5^; 0.120^7^; 0.62^8*^; 0.051^9^; 1.128^10^
	Cement line	${\mathcal{G}}_{c,\text{cem}}$		0.146^4^; 0.1629^6^;0.1629^8*^; 0.084^7^; 0.05^5,9^; 0.564^10^

Experimental studies: ^a^Faingold et al. (2014); ^b^Farley et al. (2022); ^c^Rho et al. (1999); ^d^Rho et al. (2002); ^e^Mullins et al. (2009); ^f^Zhou et al. (2020); ^g^Yadav et al. (2021); ^h^Hengsberger et al. (2002); ^i^Skedros et al. (2005); ^j^Milanovic et al. (2018); ^k^Burr et al. (1988); ^l^Montalbano and Feng (2011); ^m^Norman et al. (1995); ^n^Koester et al. (2008); ^o^Zimmerman et al. (2009); ^p^Chan et al. (2009); ^q^Nalla et al. (2005)

Numerical studies: ^1^Budyn and Hoc (2007); ^2^Budyn et al. (2008); ^3^Mishinski and Ural (2011); ^4^Abdel-Wahab et al. (2012); ^5^Demirtas et al. (2016); ^6^Giner et al. (2017); ^7^Wang et al. (2020); ^8^Maghami et al. (2021, 2022); ^9^Demirtas et al. (2023); ^10^Shin et al. (2024). (Values marked by ^*^ are from a phase-field study.)

**Table 2:** Baseline material parameters used in the designs of experiments

Parameter	Value (unit)
${E}_{\text{mat}}$	15 GPa
${E}_{\text{ost}}$	13.5 GPa
${E}_{\text{cem}}$	15 GPa
${\mathcal{G}}_{c,\text{mat}}$	0.35 N/mm
${\mathcal{G}}_{c,\text{ost}}$	0.5 N/mm
${\mathcal{G}}_{c,\text{cem}}$	0.1 N/mm

### Bovine bone experiments

All experiments were performed by Gustafsson et al. (2024) and a more comprehensive account of the experiments can be found there. Briefly, SENB specimens of dimensions 2 × 4 × 25 mm were cut from a bovine femur and a notch (~ 1.5 mm) was created using a diamond saw and sharpened with a scalpel. The specimens of interest for this study are the radial specimens; the microstructure is relatively constant along the long axis of the bone which makes these specimens suitable for 2D modelling. A total of 10 radial specimens were tested using a three-point bend setup with a span of 16 mm. The position of the crack tip and the crack-mouth opening displacement (CMOD) were tracked using digital image correlation, and force was monitored continuously. The J-integral was analysed according to ASTM E1820 (2018).^4^ The J-integral was evaluated for each time point where there was an increase in the crack length, to give the crack growth resistance curve (R-curve). Crack initiation toughness ${J}_{Ic}$ was reported as the value of the J-integral when the crack extension was 0.2 mm (cf. ASTM E1820 2018). Toughness was alternatively reported as ${J}_{Pmax}$, in which toughness is evaluated according to ASTM E399 (2006). In particular, unstable fracture was assumed to occur at peak force, with zero crack extension beforehand (cf. Ritchie et al. 2008). A subset of 6 radial specimens was subsequently imaged in micro-X-ray computed tomography (µCT) to analyse the crack tortuosity. The crack was isolated and skeletonised, and the total crack length was calculated as half the circumference of the skeletonised crack (See Supplementary material S1). Only the first 25% of the initially intact ligament height, for which the ASTM E1820 (2018) is valid, were considered. This length was normalised by the vertical crack extension to get the tortuosity for each slice in the µCT stack. Two of the imaged specimens were excluded from the simulation study. In one, the crack path varied considerably through the sample thickness, so that a representative 2D slice could not be selected, whereas in the other, the crack followed a path almost orthogonal to the initial notch, and the initial fracture toughness was very low (${J}_{Ic}=$ 0.11 N/mm). A summary of the results in terms of peak force, tortuosity and initiation toughness for the four imaged radial specimens that were selected for this study is given in Table 3.

**Table 3:** Specimens used in the numerical experiments along with specimen-specific height $h$ and notch depth ${a}_{0}$ and experimental results in terms of peak force, tortuosity and initiation toughness

Specimen	Simulation	Height ${h}$ (mm)	Notch depth ${a}$ (mm)	Exp. force (N)	Exp. tort	Exp. ${J}_{Ic}$ (N/mm)
1	Resp. surface	4.0	1.7	33	1.25	0.35
2	Validation	4.0	1.7	25	1.21	0.26
3	Validation	4.0	1.4	43	1.24	0.35
4	Validation	3.8	1.8	35	1.24	0.54

### Screening analysis

**Fig. 2:**
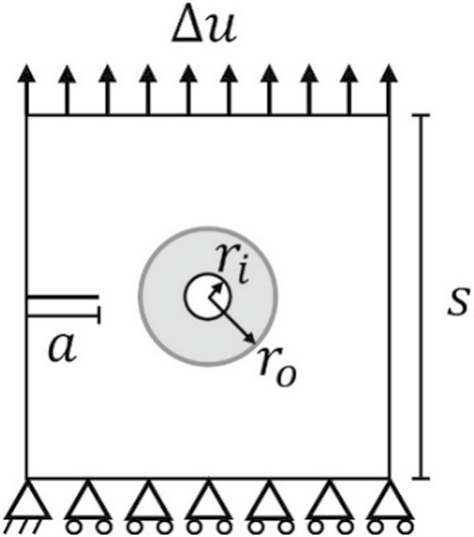
Geometry and the boundary conditions for the single-osteon model, consisting of a square of side $s=400$ µm, containing a circular osteon of outer radius ${r}_{o}=75$ µm and canal radius ${r}_{i}=25$ µm surrounded by a cement line of thickness ${t}_{\text{cem}}=5$ µm. The size of the osteon, canal and cement line are representative for osteons in both human and bovine bone (Britz et al. 2009; Budyn and Hoc 2007; Hillier and Bell 2007). A notch of length $a=80$ µm was placed at specimen mid-height. The specimen was subjected to a prescribed vertical displacement on the top face while the bottom face was constrained in the vertical direction

An initial three-level screening analysis was performed to eliminate the least important parameters. Based on the identified uncertainty in moduli for interstitial matrix and osteon (Table 1), all parameters were varied ± 20% from the baseline (Table 2). A Box–Behnken design with six parameters on three levels was used, resulting in a matrix with 54 simulations, including four repeats at the baseline (see Supplementary material S2). A simple single-osteon model subjected to uniaxial strain was considered, as shown in Fig. 2. A similar model was previously considered by Gustafsson and Isaksson (2022). The model was discretised with 40 000 first-order, four-node plane strain elements. The regions of different material properties were perfectly bonded in the sense that they shared nodes at the boundary. A fine mesh of element side length $h=1.25$ µm was used in areas where the crack was expected to propagate. The length scale parameter was chosen as ${\ell}=0.5 {t}_{cem}=2.5$ µm, as Gustafsson and Isaksson (2022) showed that this leads to negligible errors in energy release in the interface. The choices of length scale parameter and element edge length satisfies the relation $h\le {\ell}/2$ (Miehe et al. 2010). The model was loaded in displacement control using a monolithic solver and a step size of $\Delta u=5\cdot {10}^{-5}$ mm.

Post-processing of the simulation results was performed automatically using python scripting in Abaqus. Peak force was taken as the maximum force experienced, except in cases where the crack reinitiated in the canal. In these cases, the force history up to the point when the crack entered the canal was considered, since the model overpredicts strength at crack initiation due to the small length parameter ${\ell}$. Energy release per unit crack advance was taken as the accumulated change in strain energy divided by the projected crack advance in horizontal direction. For cracks passing through the osteon, only strain energy and crack advance up to the point when the crack entered the canal was considered. Tortuosity was evaluated as half of the circumference of a skeletonised damage contour plot normalised by the projected horizontal crack length.

The importance of each parameter was estimated using analysis of variance (ANOVA). The individual sums of squares for the deviation caused by the different factors (${\text{SS}}_{F}$) were calculated using Type II sums of square in Matlab^5^ using the “anovan” function. The proportion of the total sum of squares (${\text{SS}}_{T}$) of the deviation that is accounted for by each factor, $\text{TSS}={\text{SS}}_{F}/{\text{SS}}_{T},$ was considered as a measure of the relative importance of that factor.

### Response surface analysis

A response surface design was used to calibrate material parameters for the three types of microstructural tissue. A Box–Behnken design was used with a subset of four parameters (${E}_{\text{cem}}{, \mathcal{G}}_{c,\text{mat}}, {\mathcal{G}}_{c,\text{ost}}$ and ${\mathcal{G}}_{c,\text{cem}}$) at three levels, resulting in a matrix with 27 simulations including three repeats at the baseline (see Supplementary material S2). Due to the large uncertainty of the baseline, the identified parameters in Table 2 were varied ± 50% from the baseline.

**Fig. 3:**
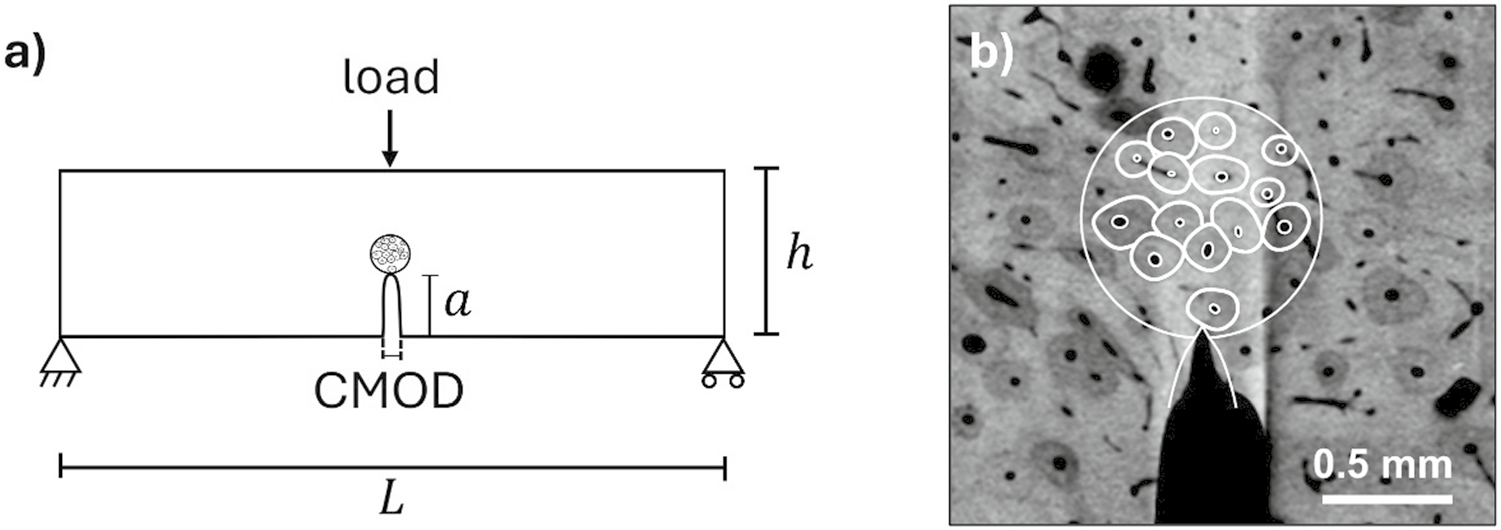
**a** The SENB model, consisting of a centrally notched beam of length $L=160$ mm, height $h\approx 4$ mm and notch depth $a\approx 1.5$ mm (cf. Table 3), with a thickness of $t=2$ mm. The model was constrained in horizontal and vertical directions in the lower left corner, and in the horizontal direction in the lower right corner. The crack-mouth opening displacement (CMOD) was used to compare with experimental results. **b** µCT image of Specimen 1, showing the manually segmented embedded cell ahead of the notch

The simulation mimicked the SENB experimental setup used by Gustafsson et al. (2024) (Fig. 3), to allow for quantitative comparisons. Specimen-specific height and notch depth were used (Table 3). A circular area ahead of the notch was segmented from µCT scans of the specimens used by Gustafsson et al. (2024). The µCT stack (voxel size 6.5 µm) was 3D mean filtered using a filter size of 2 × 2 × 6 (the larger value applies to the longitudinal direction) to reduce noise. Brightness and contrast were adjusted to enhance the contrast between osteons and matrix. An interior slice was chosen in which neither microstructure nor final crack path changed much with depth. The chosen slice was hand-segmented in Abaqus CAE using the sketcher tool by direct tracing of the osteons in the µCT image. A circle of radius 0.9 mm was considered as the area of interest. As cement lines could not be segmented from the µCT images, each osteon was assumed to be surrounded by a 5 µm thick cement line. This is in line with previous research that reports a cement line thickness of 1–5 µm (Skedros et al. 2005; Burr et al. 1988). Additionally, a cement line thickness of 5 µm is commonly used in computational studies since it allows for larger element size and hence speeds up simulations (cf. e.g. Budyn et al 2008; Gustafsson et al. 2019a; Maghami et al. 2021). Osteons and cement line were assigned the phase-field UEL with tissue-specific material parameters. The interstitial matrix material inside the circle was assigned the phase-field UEL, while the material outside the circle was assumed to be linear elastic with the same properties as the interstitial matrix but without phase-field damage. Again, the regions of different material properties shared nodes at the boundary.

The model was discretised with 200 000 first-order, four-node plane strain elements. In the area ahead of the notch, where the crack was expected to propagate, the element side length was $h=1.25$ µm. Again, a length scale parameter ${\ell}$ of 2.5 µm was used which satisfies $h\le {\ell}/2$ and leads to negligible errors in energy release in the interface. A prescribed displacement was applied on the top surface, mid-span, with a step size of $\Delta u={10}^{-4}$ mm, reducing to $\Delta u={10}^{-5}$ mm after the first drop in load, using a monolithic solver. The element edge length and displacement increments were chosen based on convergence studies (see Supplementary material S3).

When evaluating the response surface experiments, three outcomes were considered: maximum force, crack tortuosity and fracture toughness. Again, post-processing of the simulation results was performed automatically using python scripting in Abaqus. Maximum force was taken as the peak force in the force history, unless when the crack had passed through an osteon, in which case only the force history up to the point when the crack entered the canal was considered. Tortuosity was calculated as half of the circumference of a skeletonised damage contour plot normalised with the vertical distance (see Supplementary material S1). Toughness was evaluated by calculating the K-value for the entire force ${P}_{i}$ versus crack length ${a}_{i}$ history,

4 $${K}_{i}=\frac{{P}_{i}L}{t{h}^{3/2}}f\left(\frac{{a}_{i}}{h}\right),$$

where $f({a}_{i}/h)$ is a geometric correction factor given in, for example ASTM E1820 (2018) (see Fig. 3 for definition of $L, h$). The toughness value at the onset of unstable crack propagation is ${K}_{c}$. Toughness was reported in terms of the J-integral with ${J}_{Ic}={K}_{c}^{2}(1-{\nu }^{2})/E$, due to linear elasticity.

ANOVA was used to identify the most significant parameters (${\mathcal{G}}_{\text{mat}}$ and ${\mathcal{G}}_{\text{cem}}$). Linear response surfaces were fitted for these parameters, for the outcomes peak force, tortuosity and ${J}_{Ic}$, using the function “regstats” in Matlab. The error for each point on the response surface was obtained by subtracting the experimental results for Specimen 1 (Table 3) from the respective response surface. The sum of the absolute values of the error in peak force, tortuosity and ${J}_{Ic}$, normalised by the respective experimental result, was used as the cost function and the best parameter set was regarded as one that minimised the sum of the absolute errors.

### Validation

**Fig. 4:**
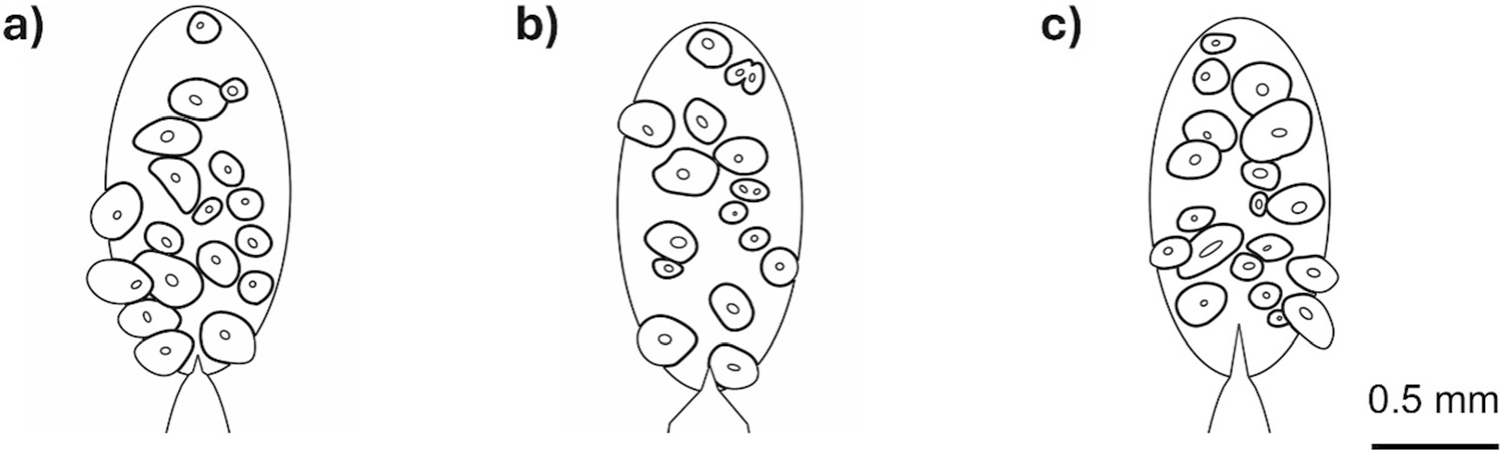
Simulation geometries for **a** specimen 2, **b** specimen 3 and **c** specimen 4 which were used to evaluate the performance of the model. The scale bar applies to all three geometries

To evaluate the performance of the model, FE predictions for three new bone specimens were compared to the experimental results from Gustafsson et al. (2024). Again, the SENB geometry was used (Fig. 3). The three new regions of interest are shown in Fig. 4, and geometry-specific height and notch depth were used (Table 3). The creation of the models was similar to the process described in Sect. 3.1. A larger region of interest—an ellipse of long axis 1.5 mm and short axis 0.75 mm—was used so that the crack could propagate without interfering with the purely linear-elastic (no phase-field damage) material. The models were discretised with about 400 000 first-order, four-node plane strain elements of edge length 1.25 µm where the crack was expected to propagate. The length parameter was again set to ${\ell}=2.5$ µm. The simulations were post-processed as described in Sect. 3.4.

## Results

### Screening analysis

ANOVA was used to identify the most important parameters for three outcomes (peak force, tortuosity and energy released per unit crack advance) in the screening experiment (Table 4). No interactions were considered. It is noted that ${\mathcal{G}}_{c,\text{mat}}$ contributed greatly to the maximum force (67% of the total variability) and energy released per unit crack advance (53%). ${\mathcal{G}}_{c,\text{ost}}$ contributed to the crack length (29%). ${\mathcal{G}}_{c,\text{cem}}$ contributed to both crack length (29%) and fracture energy (13%). ${E}_{\text{mat}}$ contributed primarily to the maximum force (26%). ${E}_{\text{ost}}$ did not give a large contribution to any outcome. Due to the relatively small contributions from moduli for matrix and osteon, combined with the fact that these parameters are comparatively well documented in the experimental literature, these were not considered in the response surface analysis. Finally, ${E}_{\text{cem}}$ did not give a large contribution to any of the outcomes considered, with a 6% contribution to crack length and 3% contribution to fracture energy. However, due to the uncertainty associated with the modulus of the cement line, it was nevertheless kept for further analysis in the response surface experiment. Phase-field damage contours and force versus displacement curve for the baseline parameters can be found in Supplementary materials S4.

**Table 4:** Importance (TSS) of each factor in the scree﻿ning analysis

Parameter	Peak force	Tortuosity	Energy release/crack advance
${E}_{\text{mat}}$	0.26^*^	0.03	0.09^*^
${E}_{\text{ost}}$	0.06^*^	0.10^*^	0.01
${E}_{\text{cem}}$	0.00^*^	0.06^*^	0.03^*^
${\mathcal{G}}_{c,\text{mat}}$	0.67^*^	0.05^*^	0.53^*^
${\mathcal{G}}_{c,\text{ost}}$	0.00	0.29^*^	0.01
${\mathcal{G}}_{c,\text{cem}}$	0.00	0.29^*^	0.13^*^
Total:	0.99	0.81	0.80

Statistically significant contributions based on ANOVA (*p* < 0.05) are indicated with*. Contributions of more than 5% of the total variability of an outcome are underlined. The parameters considered in the response surface study are in bold

### Response surface analysis

ANOVA was again used to identify significant contributions (Table 5). Main effects and interactions were considered. Almost all variability was accounted for by the main effects, and interactions contributed at most to 6% of the total variability. The greatest contribution to all outcomes was from ${\mathcal{G}}_{c,\text{cem}}$, which contributed to peak force (52% of total variability), tortuosity (38%) and toughness (61%). Also ${\mathcal{G}}_{c,\text{mat}}$ contributed to all outcomes, with the largest contribution to crack length (24%). ${E}_{\text{cem}}$ contributed to the tortuosity (13%) and to some extent to ${J}_{Ic}$ (7%), with no significant contribution to maximum force. The contributions from ${\mathcal{G}}_{c,\text{ost}}$ were generally small. In addition to giving the greatest contributions out of the main effects, the only significant contribution from interactions was from the interaction between ${\mathcal{G}}_{c,\text{mat}}$ and ${\mathcal{G}}_{c,\text{cem}}$. Phase-field damage contours and curves of both force and crack extension versus CMOD for the baseline parameters can be found in Supplementary material S5.

**Table 5:** Importance (TSS) of each factor in the response surface analysis. Contributions of more than 5% of the total variability of an outcome are underlined. The most important parameters, for which response surfaces are fitted, are marked in** bold**

Parameter	Peak force	Tortuosity	${J}_{Ic}$
${E}_{\text{cem}}$	0.03	0.13	0.07
${\mathcal{G}}_{c,\text{mat}}$	0.19	0.24	0.15
${\mathcal{G}}_{c,\text{ost}}$	0.00	0.05	0.00
${\mathcal{G}}_{c,\text{cem}}$	0.52	0.38	0.61
${E}_{cem}\cdot {\mathcal{G}}_{c,\text{mat}}$	0.00	0.00	0.01
${E}_{\text{cem}}\cdot {\mathcal{G}}_{c,\text{ost}}$	0.00	0.00	0.00
${\mathcal{G}}_{c,\text{mat}}\cdot {\mathcal{G}}_{c,\text{ost}}$	0.00	0.00	0.03
${E}_{\text{cem}}\cdot {\mathcal{G}}_{c,\text{cem}}$	0.00	0.03	0.00
${\mathcal{G}}_{c,\text{mat}}\cdot {\mathcal{G}}_{c,\text{cem}}$	0.06	0.02	0.01
${\mathcal{G}}_{c,\text{ost}}\cdot {\mathcal{G}}_{c,\text{cem}}$	0.00	0.00	0.00
Total	0.81	0.85	0.90

**Fig. 5:**
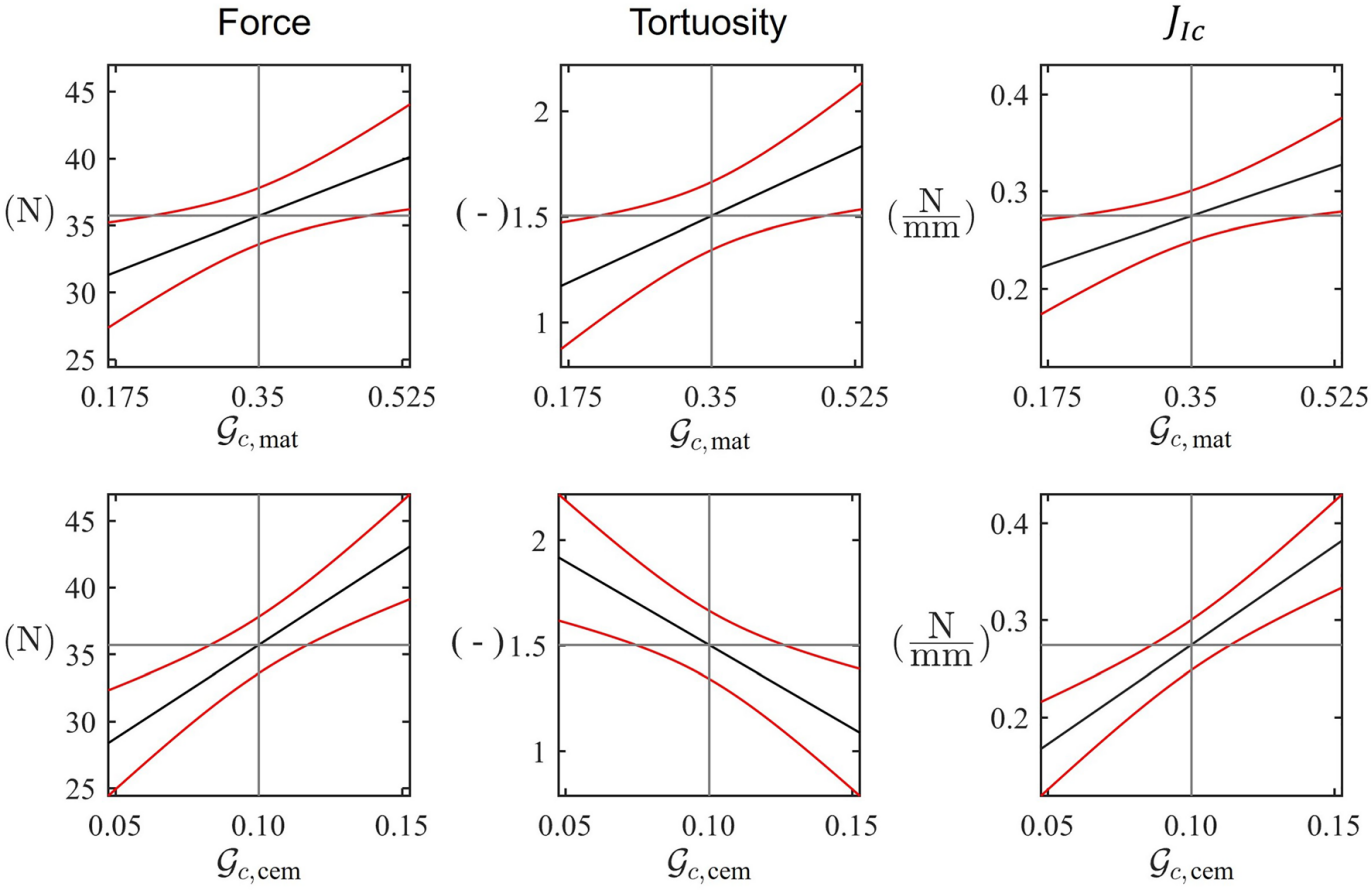
Response surfaces for force, tortuosity and ${J}_{Ic}$ with 95% confidence intervals. Each surface is shown as two orthogonal cuts to show dependence upon for parameters ${\mathcal{G}}_{c,\text{mat}}$ (*top row*) and ${\mathcal{G}}_{c,\text{cem}}$ (*bottom row*)

Since the variability is dominated by two parameters, ${\mathcal{G}}_{c,\text{mat}}$ and ${\mathcal{G}}_{c,\text{cem}}$, these were the only parameters considered when fitting response surfaces. The prediction profiles of the two parameters are shown at the baseline values in Fig. 5. The minimum error between the normalised response surfaces and the experimental results (Table 3) occurs at ${\mathcal{G}}_{c,\text{mat}}=0.36$ N/mm and ${\mathcal{G}}_{c,\text{cem}}=0.14$ N/mm.

### Validation

**Fig. 6:**
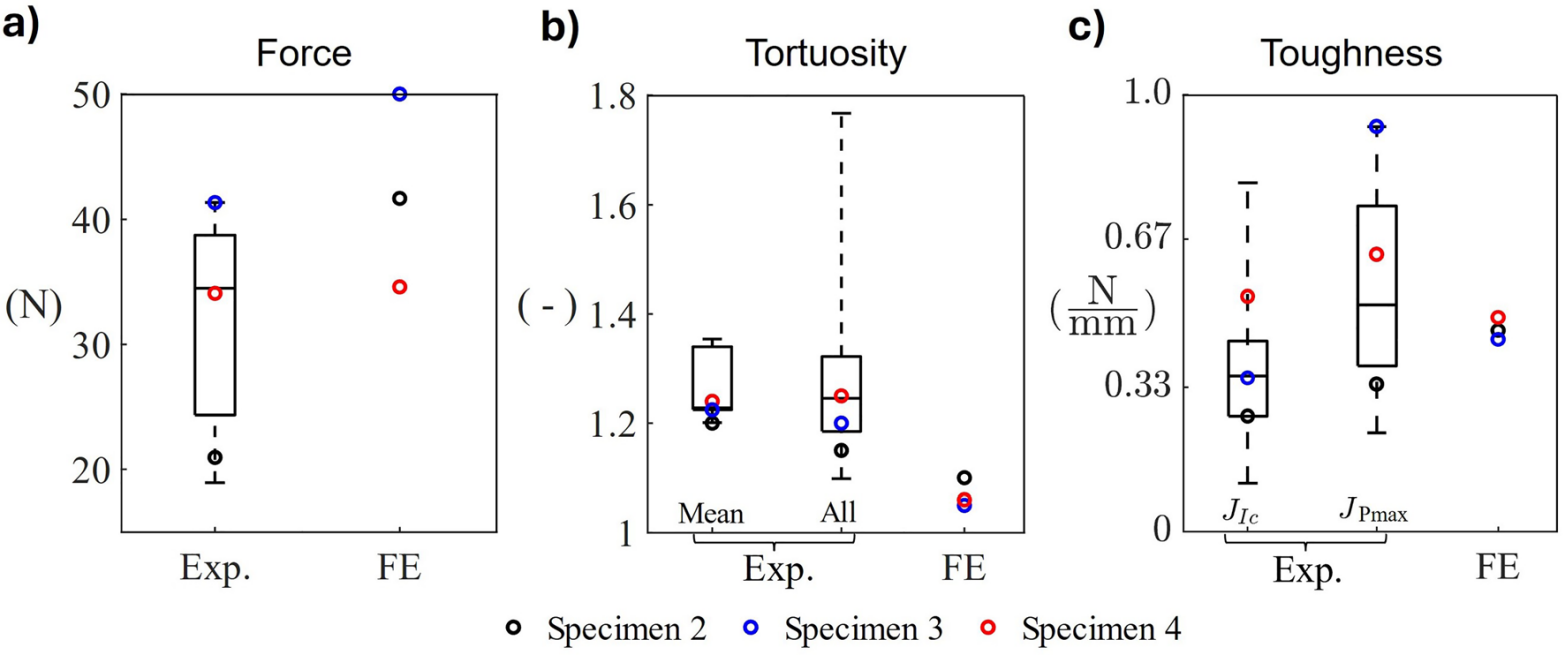
Comparison of FE predictions obtained with final material parameters (Table 6), to box plots of experimental results for **a** force (${N}_{\text{Exp}}=10$), **b** tortuosity (${N}_{\text{Exp}}=6$) in terms of both mean tortuosity per specimen and tortuosity per image slice (~ 300 images per specimen), and **c** fracture toughness (${N}_{\text{Exp}}=10$) in terms of both ${J}_{Ic}$ and ${J}_{\text{Pmax}}$. The box plots indicate (from bottom to top) minimum value, 25-percentile, median, 75-percentile and maximum value. Individual experimental results for the three simulated specimens (in the case of per-slice tortuosity for the exact slice used for simulation) are indicated with *circles*

When comparing the FE predictions, using the properties in Table 6, to the experiments of Gustafsson et al. (2024), the predicted force was in general 0–50% too high, but all except one predicted data point fell within the variability of the experiments (Fig. 6a). Notably, the predicted highest force was for the specimen that also exhibited the highest force experimentally. Tortuosity was generally underpredicted in simulations (Fig. 6b), while toughness was reasonably well predicted, both with respect to ${J}_{Ic}$ and ${J}_{\text{Pmax}}$ (Fig. 6c).

**Table 6:** Material properties used in the validation. Values marked in bold were updated from their baseline value following the response surface analysis

Parameter	Value (unit)
${E}_{\text{mat}}$	15 GPa
${E}_{\text{ost}}$	13.5 GPa
${E}_{\text{cem}}$	15 GPa
${\mathcal{G}}_{c,\text{mat}}$	**0.36 N/mm**
${\mathcal{G}}_{c,\text{ost}}$	0.5 N/mm
${\mathcal{G}}_{c,\text{cem}}$	**0.14 N/mm**

**Fig. 7:**
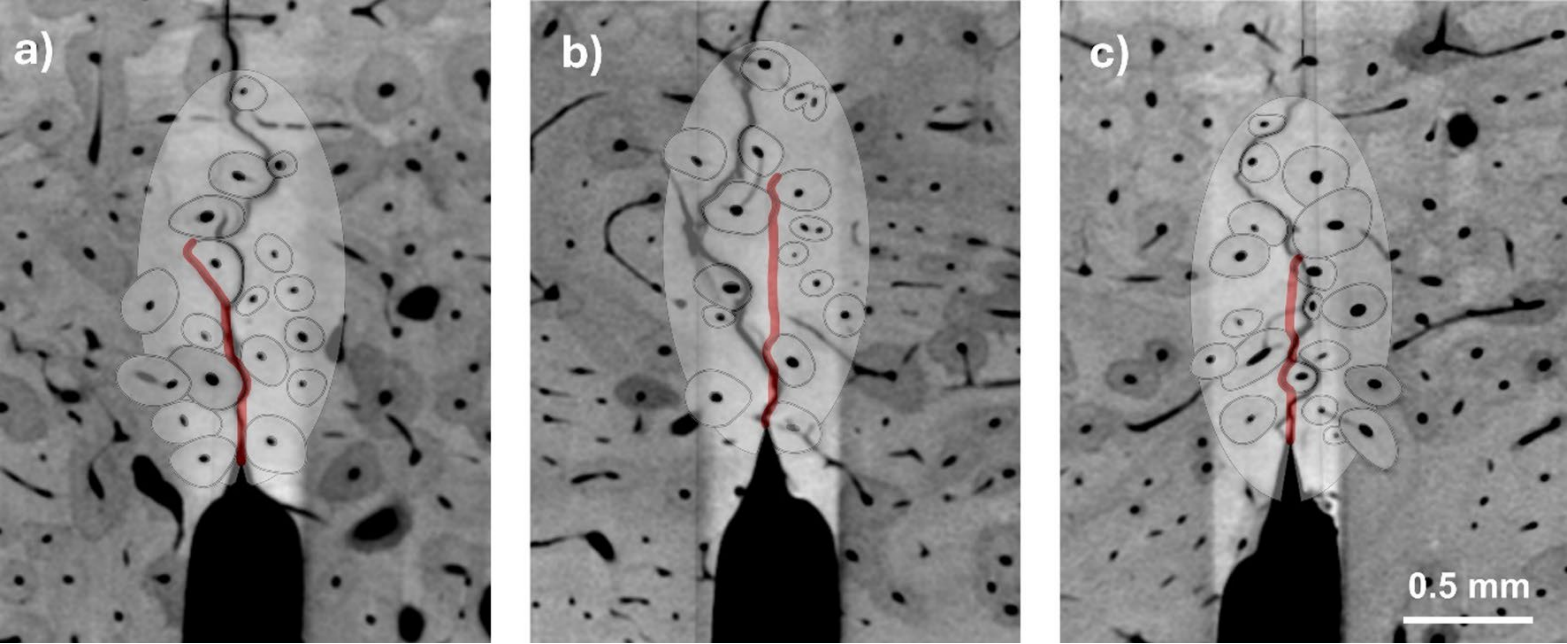
Crack paths ($d>0.01$) obtained in validation simulations overlaid on the corresponding post-experiment µCT images for **a** specimen 2, **b** specimen 3 and **c** specimen 4 with material parameters given in Table 6. Scalebar applies to all figures

The resulting crack paths are shown overlaid with post-experiment µCT scan images of the corresponding specimens (Fig. 7). In all three cases, the simulated crack propagated predominantly in the vertical direction with occasional deflections in cement lines. In contrast, the experimental cracks display a more tortuous crack path, with cracks appearing to be affected by features that were not included in the simulation model.

**Fig. 8:**
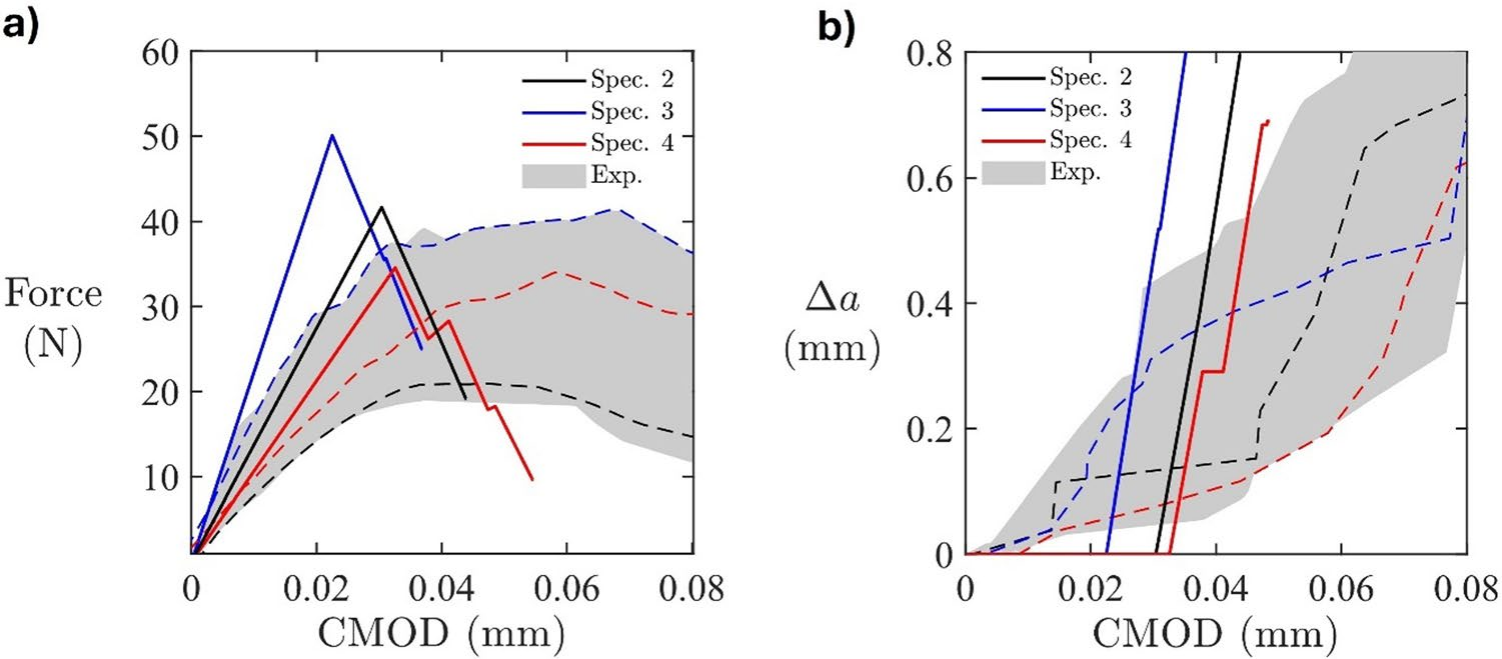
Predictions of **a** force versus CMOD and **b** crack extension $\Delta a$ versus CMOD curves for the specimens 2, 3 and 4. The shaded areas indicate the full range of experimental results (${N}_{\text{Exp}}=10$); the experimental curves for specimens 2, 3 and 4 are indicated with dashed lines

It is remarkable, given the overall acceptable agreement between prediction and experiment evidenced by Figs. 6 and 7, that the overall force versus CMOD response was not well captured (Fig. 8). In particular, the deformation near and after the peak load is underpredicted. Simulations predict a linear-elastic response, with a stiffness which is within the experimental envelope. However, the experimental response is nonlinear, and the simulations hence underpredict the deformation observed in experiments. Notably, the experiments exhibit small amount of crack growth already from a very low CMOD (or low force), unlike simulations. The decrease in load-carrying ability is accompanied by an increase in crack growth rate for both simulation and experiment, but in experiments, the rate of crack growth remains moderate also after this, unlike the simulations.

## Discussion

A problem associated with all modelling work on bone microstructure is the lack of material parameters, which in turn is associated with the difficulty of measuring properties on this scale. To address this issue, the present study employed a DOE approach to finding suitable values for material parameters needed for phase-field simulation of fracture in cortical bone. Moreover, with few material parameters available, models need to be simple; in this regard classical, linear-elastic, brittle phase-field models like the ones employed by Maghami et al. (2021; 2022) and Josephson et al. (2022) appear ideal as they require very few parameters. But, until now, it has not been clear to what extent the predictions of phase-field models of cortical bone can be trusted, as validation to experimental data has not previously been done. Thus, following parameter identification, the model was closely compared with experimental data, both in terms of mechanical data, and in terms of the predicted crack paths.

First of all, a literature study was conducted to find baseline values for the six relevant parameters (Table 2). In a screening study, all six material parameters were varied to determine their sensitivity. Out of the six parameters, five were seen to affect at least one of the evaluated outcomes. Among the five were the moduli of the interstitial matrix and osteons, which were nevertheless excluded from further analysis. The reason for this is twofold. First, the contributions were not great, and second, the moduli of these tissue types were the only parameters for which sufficient experimental data exists (Table 1). Considering that these moduli reasonably ought to be varied by less than the other parameters due to the smaller uncertainty, the relative importance of these moduli is deemed small. There is a risk that this choice may have affected the calibrated values of ${\mathcal{G}}_{c,\text{mat}}$ and ${\mathcal{G}}_{c,\text{cem}}$; however, the initial stiffnesses of the simulated and experimental results are in good agreement (Fig. 8), indicating that the moduli are indeed reasonable.

The response surface analysis, using the SENB geometry employed in the experimental study by Gustafsson et al. (2024) indicates that the toughness of the matrix and the cement line are the most important material parameters. The toughness found for the cement line is in line with the value found by Giner et al. (2017) and thus appears to confirm the hypothesis that the cement line has considerably lower toughness than the surrounding tissue. The findings also support the idea put forth in Gustafsson and Isaksson (2022) that the relation between cement line toughness and that of the surrounding tissue determines the crack path.

**Fig. 9:**
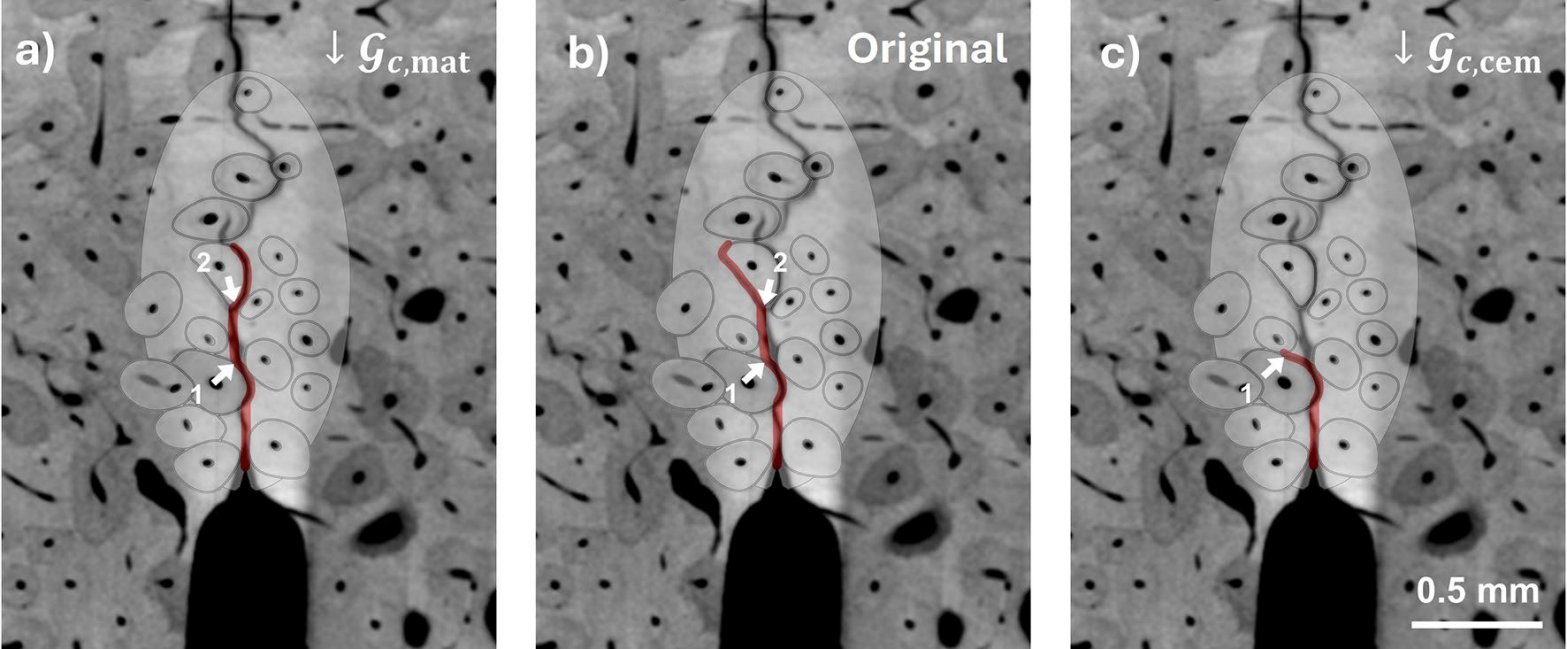
Crack path ($d>0.01$) sensitivity to variations in ${\mathcal{G}}_{c,\text{mat}}$ and ${\mathcal{G}}_{c,\text{cem}}$, illustrated for the case of specimen 2 with **a** reduced ${\mathcal{G}}_{c,\text{mat}}$ (i.e. ${\mathcal{G}}_{c,\text{mat}}=0.35$ N/mm, ${\mathcal{G}}_{c,\text{cem}}=0.14$ N/mm), **b** with the original parameters (${\mathcal{G}}_{c,\text{mat}}=0.36$ N/mm, ${\mathcal{G}}_{c,\text{cem}}=0.14$ N/mm) and **c** with reduced ${\mathcal{G}}_{c,\text{cem}}$ (i.e. ${\mathcal{G}}_{c,\text{mat}}=0.36$ N/mm, ${\mathcal{G}}_{c,\text{cem}}=0.13$ N/mm). Other material parameters are given in Table 6. Scalebar applies to all figures

In terms of the validation study, it should be noted that a perfect agreement in terms of crack paths might not be reasonable to expect given the simplified modelling (2D plane strain), and not least when considering that the crack path is highly sensitive to small variations in material parameters, as illustrated in Fig. 9. A small decrease in ${\mathcal{G}}_{c,\text{mat}}$ of 0.01 N/mm (Fig. 9a) leads to the crack leaving the cement line (indicated with 1) somewhat earlier than in the original simulation (Fig. 9b) which leads to differences later in the simulation where the crack deflects right instead of left at the next osteon (indicated with 2). On the other hand, a small decrease in ${\mathcal{G}}_{c,\text{cem}}$ of 0.01 N/mm (Fig. 9c) makes propagation in the cement line easier and the crack remains in the cement line for longer. It is noted that these small differences in material properties do not affect peak force and toughness (see Supplementary materials S6). Despite the imperfect agreement between simulated and experimental crack paths, the damage mechanism of deflection in cement lines is captured, and both Figs. 7 and 9 show qualitative agreement between experimental and simulated crack paths.

**Fig. 10:**
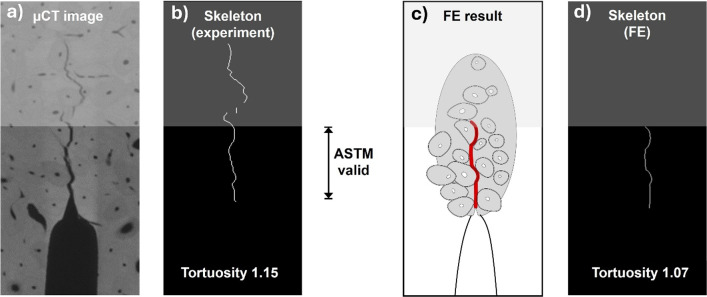
Difference in tortuosity between **a**, **b** experiment and **c**, **d** simulation. The experimental crack in (**a**), evaluated as the length of the skeletonised crack (**b**), has a tortuosity in the valid region of 1.15. The predicted crack with reduced ${\mathcal{G}}_{c,\text{mat}}$ (i.e. ${\mathcal{G}}_{c,\text{mat}}=0.35$ N/mm and ${\mathcal{G}}_{c,\text{cem}}=0.14$ N/mm) in (**c**), evaluated the same way by taking the circumference of the skeletonised crack (**d**) has a tortuosity of 1.07

In general, in the validation simulations, the maximum force is somewhat overpredicted but, given the variability within experiments, still acceptable. This is also the case for the toughness. The tortuosity, on the other hand, is generally predicted too low compared to experiments. In part, this is explained by the smooth nature of the phase-field crack, which means that even when crack paths are in excellent agreement, the tortuosity differs between simulation and experiments. Figure 10 illustrates the difference in tortuosity between experiment and simulation for a simulation which predicted a crack path in excellent agreement with the experimental one.

The higher tortuosity estimated in experiments may be attributed to the sub-microstructure of the bone tissue, which produces jagged crack paths within and across the layers of aligned mineralised collagen fibrils. The orientation of collagen fibrils at the cement line, with most of the fibres oriented along rather than across the cement line (Raguin et al. 2021), might explain that cracks appear smoother when following a cement line compared to when propagating through interstitial matrix (cf. Figure 10), and also the low value of cement line toughness identified in the response surface analysis. The simulation model, on the other hand, assumes that the material within each of the three tissue types is homogeneous and isotropic, and thus cannot account for this behaviour.

The sub-microstructure is also responsible for a large part of both the intrinsic and extrinsic toughening observed in bone (cf. Launey et al. 2010), while the phase-field model in its current form with isotropic homogeneous representation of the tissue does not accurately represent such toughening mechanisms. Intrinsic and—more significantly—extrinsic toughening may be responsible for the differences in mechanical response between simulation and experiment. Although the initial stiffness is captured by the simulation model, gradual damage accumulation in experiments leads to a nonlinear force versus CMOD response. Contrarily, the simulation model predicts a linear-elastic response up to the peak load and thus underpredicts the displacement at peak load. After the peak, the simulations predict rapid crack growth and a simultaneous decrease in load-carrying ability. The experiments instead show a gradual decrease in load-carrying ability associated with a rising R-curve. The predicted linear-elastic, brittle fracture behaviour is inherent in all classical phase-field methods and thus not unique to the current study. While phase-field models have many advantages over, for example XFEM, the cohesive formulation governing damage evolution commonly used in XFEM models of cortical bone fracture appears to agree better with the experimental data. However, phase-field methods are not restricted to the present formulation. Different crack driving source terms (i.e. $\mathcal{H}(x,t)$ in the present work) can be considered to control the speed of the crack, and the degradation function (i.e. ${\left(1-d\right)}^{2}$ in the present work) can be modified to provide a better agreement with the observed force-CMOD response. For example, intrinsic toughening may be accounted for by including plasticity in the phase-field formulation (Ambati et al. 2015b; Miehe et al. 2015). Extrinsic toughening can be represented by a cohesive response in the degradation function (Conti et al. 2016; Navidtehrani et al. 2020; Wu and Nguyen 2018), which may be a promising research direction to consider also in the field of bone micro-mechanics. Such extensions are an active research topic where the phase-field approximations are yet to be verified (Francfort 2022).

The present study has a number of limitations. First and foremost, the simulation model used in this study is in 2D. While this is common in the field of bone fracture mechanics, it limits studies to the crack propagation in the radial direction. Even in this direction, the tissue microstructure is not constant though the thickness; osteons are never perfectly parallel with the longitudinal axis, and additionally, in-plane features such as Volkmann canals might affect crack propagation. Smaller features, like cell lacunae also exist, but have been shown not to affect the crack path (Josephson et al. 2022). Moreover, the choice to use a geometric notch, as opposed to introducing the notch by prescribing phase-field damage or strain energy history, may have contributed to some overprediction of the force at crack onset (Kristensen et al. 2021). Also, since only one model was used for parameter calibration, it cannot be excluded that the identified material parameters are to some extent specimen-dependent. This effect is however believed to be small compared to other sources of uncertainty, such as variability within and between biological specimens and the linear-elastic behaviour of the phase-field model.

Future research on this topic may want to investigate how the phase-field method can be modified to give a better agreement with experimental data, not least how the decidedly nonlinear fracture behaviour of bone can be captured in such a setting. Of similar importance is work with developing computationally efficient 3D models (cf. Demirtas et al. 2023). The present 2D models are restricted to simulating crack propagation in the radial direction of the bone, whereas the more physiologically relevant direction is the transverse (breaking) fracture. This likely requires a different modelling strategy for the cement line (or cement sheath as it is no longer a line in 3D), as the current model’s fine mesh makes extension to the third dimension unfeasible. Possibilities to model the cement line using cohesive zones could be explored, as well as using a strongly anisotropic surface energy near or in osteons (cf. Clayton and Knap 2015).

## Conclusions

In this study, mechanical properties for phase-field fracture simulation of cortical bone microstructure have been estimated based on quantitative comparisons between high-resolution phase-field simulations and experimental results. The analysis supports that despite only contributing a small proportion of the material, the cement line toughness plays an important role for both the notched strength of the cortical bone specimens and the ensuing crack path.

The initial stiffness, peak load and toughness are reasonably well captured by the simulation model. Even so, the linear-elastic phase-field model does not capture the force versus CMOD response of the bone specimens satisfactorily, in particular the deformation near and after the peak load is underpredicted. In experiments, damage is evolving already at low load, leading to a nonlinear force vs CMOD response and crack growth is stable with a rising R-curve. Classical phase-field methods, which rely on linear-elastic fracture mechanics, are unable to capture these features of gradual damage evolution and rising R-curve.

Validation is a crucial step in all modelling and establishes trust in a model. This is the first study that quantitatively evaluates performance of phase-field modelling for fracture of cortical bone. It highlights both potential and limitations as well as outlines the importance of further work, since accurate material properties and validated modelling approaches are required to advance the research towards understanding why damage mechanisms change between young to old bone.

## Supplementary Information

Below is the link to the electronic supplementary material.

Supplementary file1 (PDF 834 kb)

## Data Availability

Data will be made available upon reasonable request.

## Footnotes

1 Brittle is used in the fracture mechanics sense that fracture occurs with negligible plastic strain.

2 Element-deletion methods tend to be mesh-sensitive and will not be discussed further.

3 Abaqus/Standard version 2023. Dassault Systèmes Simulia Corp. Providence, RI, USA.

4 The use of J-integral for assessment of bone toughness is somewhat debated, since the J-integral assumes homogeneous materials. However, in the absence of designated standards, fracture toughness of bone is typically evaluated according to either ASTM E399 (2006) or to ASTM E1820 (2018). Out of the two, the latter is preferred as it quantifies both the elastic and plastic contributions to the energetic cost of growing a crack, as outlined in the review by Daapah et al. (2023).

5 Matlab version R2022a. Mathworks inc. Natick, MA, USA.
