# Supplementary material for: Phase-field simulation of crack growth in cortical bone microstructure: parameter identification and comparison against experiments
Source: Biomech Model Mechanobiol. 2025 Mar 1;24(2):599–613. doi: 10.1007/s10237-025-01929-8 (PMC12055898; doi:10.1007/s10237-025-01929-8)
Supplement: Supplementary file 1 — Supplementary file1 (PDF 834 kb) [file 10237_2025_1929_MOESM1_ESM.pdf]

Supplementary material for

Phase-field simulation of crack growth in cortical bone microstructure: parameter identification and comparison against experiments

Jenny Carlsson*, Olivia Karlsson, Hanna Isaksson, Anna Gustafsson
Department of Biomedical Engineering, Lund University, Box 118, 221 00, Lund, Sweden
*Corresponding author email: jenny.carlsson@bme.lth.se

https: /doi.org/10.1007/s10237-025-01929-8

Supplementary material S1: Calculation of crack tortuosity

Both experimental and simulated tortuosity was calculated from images which were processed and analysed using ImageJ[1] and Matlab[2] (Fig. S1). The procedure for calculating the tortuosity experimentally can be found in Gustafsson et al.[3]. Briefly, post-fracture images were obtained using high-resolution µCT (Zeiss Xradia, voxel size of 6.5 µm, tube voltage 60 kV). The images were binarized, and canals were identified and removed based on a circularity measure. The initial notch was removed using a mask representing the initial notch obtained from scans of untested specimens. The resulting segmented crack was subjected to a skeletonise operation, segmenting the crack into a one-pixel wide centreline. The length of the crack was calculated using the total perimeter divided by two, using built-in region properties in Matlab. In simulations, contour plots of phase-field damage were exported from Abaqus[4] using a colourmap ranging from black to white and a black background. Again, the images were binarized and skeletonised and the length of the crack was calculated as one half of the total perimeter. Tortuosity was calculated as the total length of the crack divided by the projected length of the crack in the vertical direction.

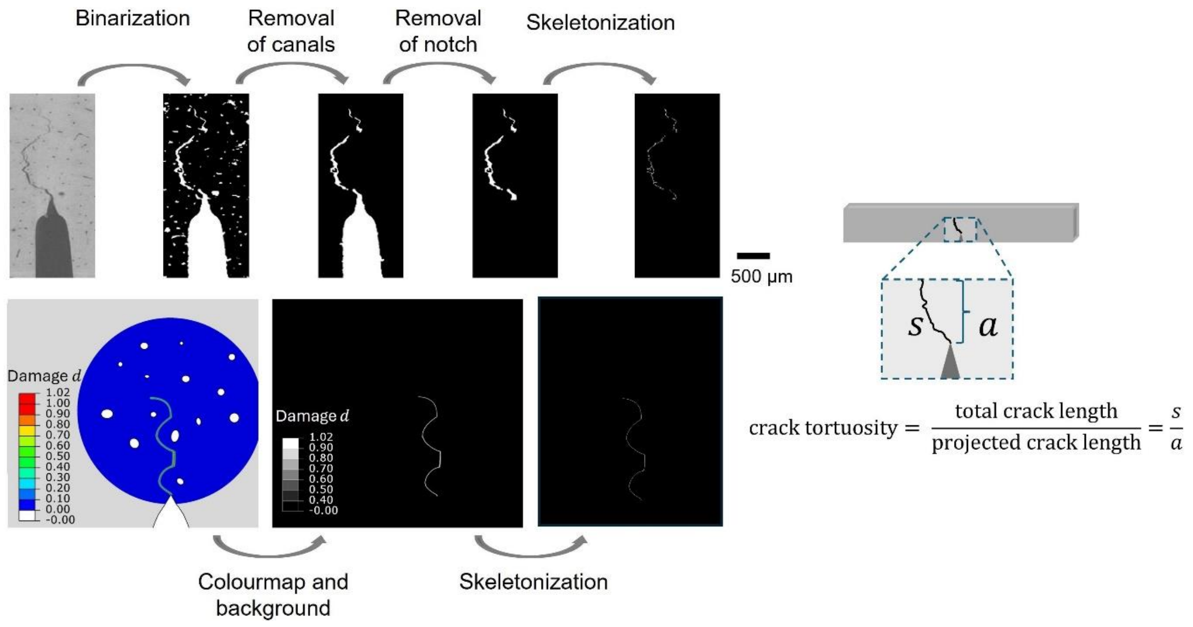

Fig. S1 Calculation of tortuosity in experiments (top row) and simulation (bottom row).

---

[1] Schneider CA, Rasband WS, Eliceri KW (2012) NIH Image to ImageJ: 25 years of image analysis. Nat Methods 9:671-675.

[2] Matlab version R2022a. Mathworks inc. Natick, MA, USA.

[3] Gustafsson A, Galteri G, Barakat A, Engqvist J, Grassi L, Cristofolini L, Dejea H, Isaksson H (2024) Characterization of damage mechanisms in cortical bone: Quantification of fracture resistance, critical strains, and crack tortuosity. J Mech Behav Biomed Mater 160:106721.

[4] Abaqus version 2023. Dassault Systèmes Simulia Corp. Providence, RI, USA.

Supplementary material S2: Box-Behnken DOE matrices for screening and response surface studies

The matrices used for screening analysis and response surface analysis are shown in Table S1 and S2, respectively.

Table S1 The matrix used for screening analysis, with 6 factors, 3 levels and 54 treatment condition

	E_{mat}	E_{ost}	E_{cem}	$\mathcal{G}_{mat}$	$\mathcal{G}_{ost}$	$\mathcal{G}_{cem}$
1	-1	-1	0	-1	0	0
2	-1	-1	0	1	0	0
3	-1	1	0	-1	0	0
4	-1	1	0	1	0	0
5	1	-1	0	-1	0	0
6	1	-1	0	1	0	0
7	1	1	0	-1	0	0
8	1	1	0	1	0	0
9	0	-1	-1	0	-1	0
10	0	-1	-1	0	1	0
11	0	-1	1	0	-1	0
12	0	-1	1	0	1	0
13	0	1	-1	0	-1	0
14	0	1	-1	0	1	0
15	0	1	1	0	-1	0
16	0	1	1	0	1	0
17	0	0	-1	-1	0	-1
18	0	0	-1	-1	0	1
19	0	0	-1	1	0	-1
20	0	0	-1	1	0	1
21	0	0	1	-1	0	-1
22	0	0	1	-1	0	1
23	0	0	1	1	0	-1
24	0	0	1	1	0	1
25	-1	0	0	-1	-1	0
26	1	0	0	-1	-1	0
27	-1	0	0	-1	1	0
28	1	0	0	-1	1	0
29	-1	0	0	1	-1	0
30	1	0	0	1	-1	0
31	-1	0	0	1	1	0
32	1	0	0	1	1	0
33	0	-1	0	0	-1	-1
34	0	1	0	0	-1	-1
35	0	-1	0	0	-1	1
36	0	1	0	0	-1	1
37	0	-1	0	0	1	-1
38	0	1	0	0	1	-1
39	0	-1	0	0	1	1
40	0	1	0	0	1	1
41	-1	0	-1	0	0	-1
42	-1	0	1	0	0	-1
43	1	0	-1	0	0	-1
44	1	0	1	0	0	-1
45	-1	0	-1	0	0	1
46	-1	0	1	0	0	1
47	1	0	-1	0	0	1
48	1	0	1	0	0	1
49	0	0	0	0	0	0
50	0	0	0	0	0	0
51	0	0	0	0	0	0
52	0	0	0	0	0	0
53	0	0	0	0	0	0
54	0	0	0	0	0	0

Table S2 The matrix used for response surface analysis, with 4 factors, 3 levels and 27 treatment conditions

	E_{cem}	$\mathcal{G}_{mat}$	$\mathcal{G}_{ost}$	$\mathcal{G}_{cem}$
1	-1	-1	0	0
2	-1	1	0	0
3	1	-1	0	0
4	1	1	0	0
5	0	0	-1	-1
6	0	0	-1	1
7	0	0	1	-1
8	0	0	1	1
9	-1	0	0	-1
10	-1	0	0	1
11	1	0	0	-1
12	1	0	0	1
13	0	-1	-1	0
14	0	-1	1	0
15	0	1	-1	0
16	0	1	1	0
17	-1	0	-1	0
18	-1	0	1	0
19	1	0	-1	0
20	1	0	1	0
21	0	-1	0	-1
22	0	-1	0	1
23	0	1	0	-1
24	0	1	0	1
25	0	0	0	0
26	0	0	0	0
27	0	0	0	0

Supplementary material S3: Convergence studies

Simulations of the osteonal microstructure are sensitive to discretisation in both space and time. To identify a converged element size and displacement increment, two convergence studies were performed. The load case considered was one of uniaxial strain (Fig. S2a). The material parameters in these simulations were E_{mat} =15 GPa, E_{ost} =12 GPa, E_{cem} =18 GPa, $\mathcal{G}_{mat}$ =0.04 N/mm, $\mathcal{G}_{ost}$ =0.48 N/mm and $\mathcal{G}_{cem}$ =0.17 N/mm. Throughout, ν =0.3 and $\ell = 0.00375$ mm. First, convergence of the solution with respect to element edge length h was analysed. We note that already $h = 0.67\ell$ is converged, even though it does not fulfil the criterion $\ell > 2h$ (Fig. S2 b-e). Second, convergence of the solution with respect to the increment of uniaxial nominal strain $\Delta\varepsilon$ was analysed, using the same simulation geometry (Fig. S3 a-d). A strain increment of $\Delta\varepsilon = 10^{-6}$ corresponds to a load-line displacement increment at the notch tip for the SENB specimen of $\Delta u = 10^{-4}$ mm. Convergence is obtained for a strain increment of $\Delta\varepsilon = 10^{-5}$.

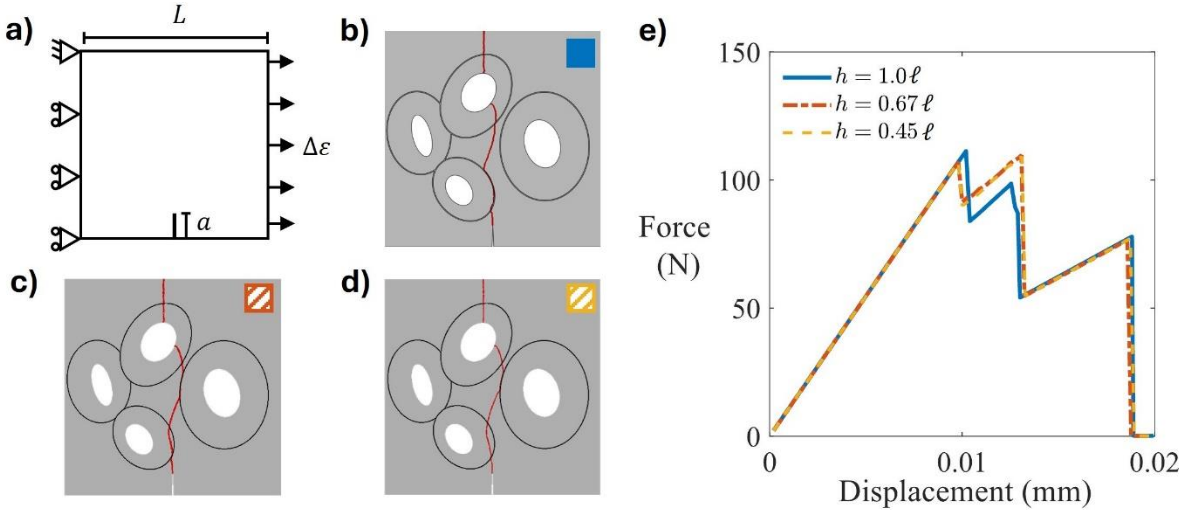

Fig. S2 Convergence with respect to element size for constant regularisation length ℓ. (a) Simulation model geometry and boundary conditions for convergence analysis, where L =1.2 mm and $a/L = 0.1$. (b)-(d) Crack paths for (b) $h = 1.0\ell$, (c) $h = 0.67\ell$ and (d) $h = 0.45\ell$. (e) Force vs displacement curves for the three different mesh refinements in (b)-(d). In all simulations above $\Delta\varepsilon = 10^{-4}$.

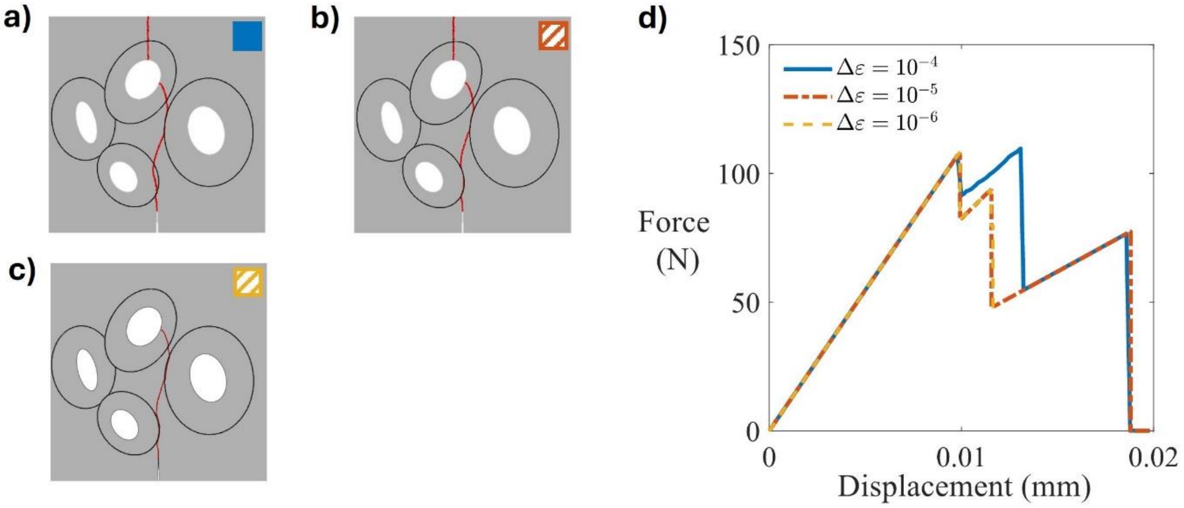

Fig. S3 Convergence of the solution with respect to the solution step $\Delta\varepsilon$. (a)-(c) Crack paths for (a) $\Delta\varepsilon = 10^{-4}$, (b) $\Delta\varepsilon = 10^{-5}$ and (c) $\Delta\varepsilon = 10^{-6}$. (d) Force vs displacement response for the three different solution steps in (a)-(c). In all simulations above $h = 0.67\ell$.

Supplementary material S4: Damage contours and curves of force vs displacement for single osteon model with baseline parameters

Damage contour plots for the single osteon model, used in the screening analysis, analysed at the baseline material parameter values (Table 2) is shown in Fig. S4(a). The width of the damaged zone is similar to the width of the cement line. The corresponding force vs displacement curve is shown in Fig. S4(b).

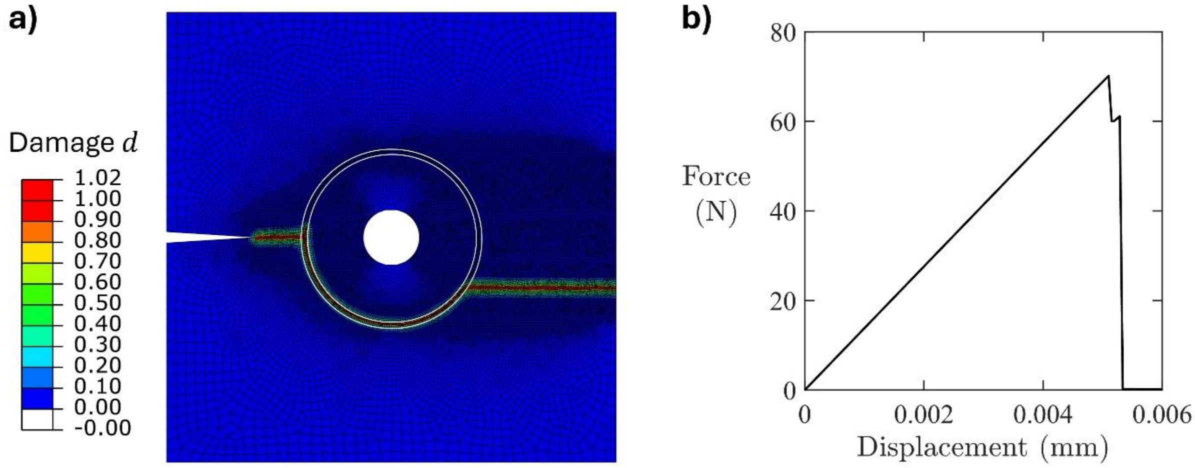

Fig. S4 Results for the specimen used in screening analysis at the baseline parameters (Table 2). (a) Damage contour and (b) force vs displacement curve.

Supplementary material S5: Damage contour and curves of force and crack extension for Specimen 1 with baseline properties

Damage contour plots for Specimen 1, used for response surface analysis, analysed at the baseline material parameter values (Table 2) are shown in Fig. S5. The width of the damaged zone is similar to the width of the cement line. The corresponding force vs CMOD and crack extension vs CMOD curves are shown in Fig. S6.

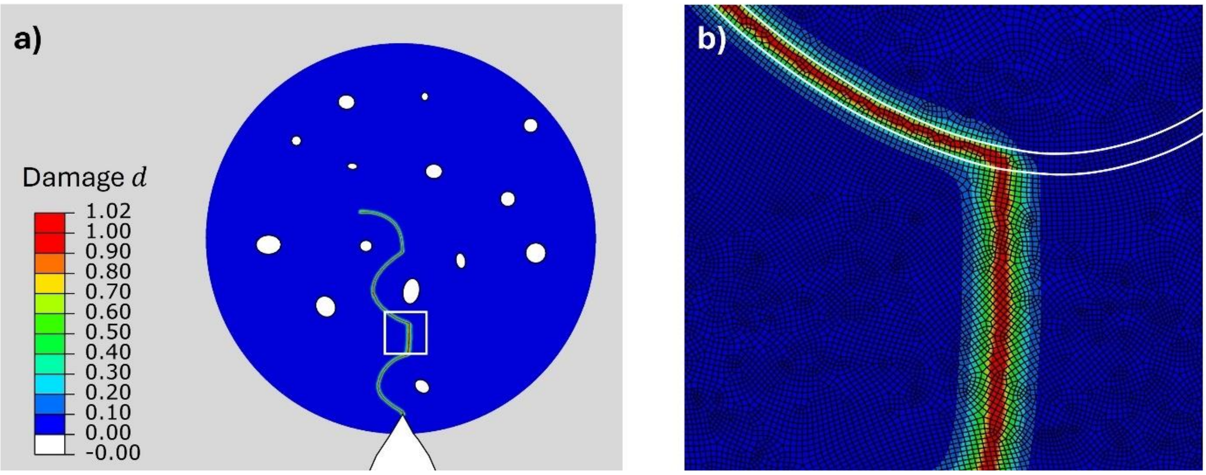

Fig. S5 Damage plot for the specimen used in response surface analysis (Specimen 1) at baseline material parameters (Table 2): (a) damage contour and (b) close-up of damage contour near a cement line showing mesh density and width of the damaged zone (crack). Colour bar refers to both figures.

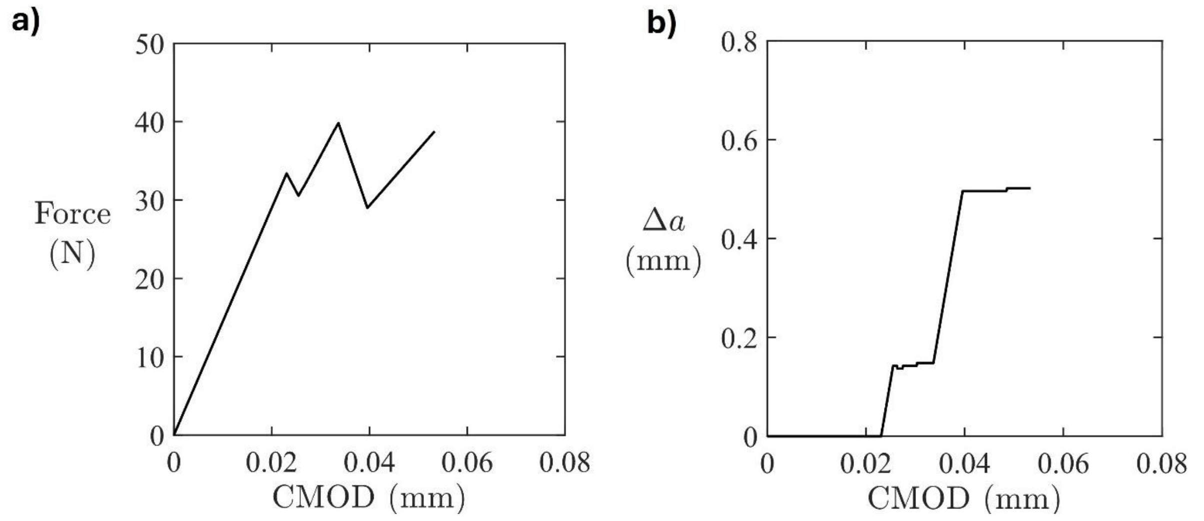

Fig. S6 (a) Force vs CMOD and (b) crack extension Δa vs CMOD curves for the specimen used in response surface analysis (Specimen 1) at baseline material parameters (Table 2).

Supplementary material S6: Force and crack extension curves for Specimen 2 with small changes in toughness

Figure S7 shows the force vs CMOD and crack extension vs CMOD curves for Specimen 2 when the fracture toughness of interstitial matrix and cement line are varied by a small amount (cf. the damage contours of Fig. 9). We note that peak force and onset of crack propagation (and consequently toughness) remain unaffected by these changes.

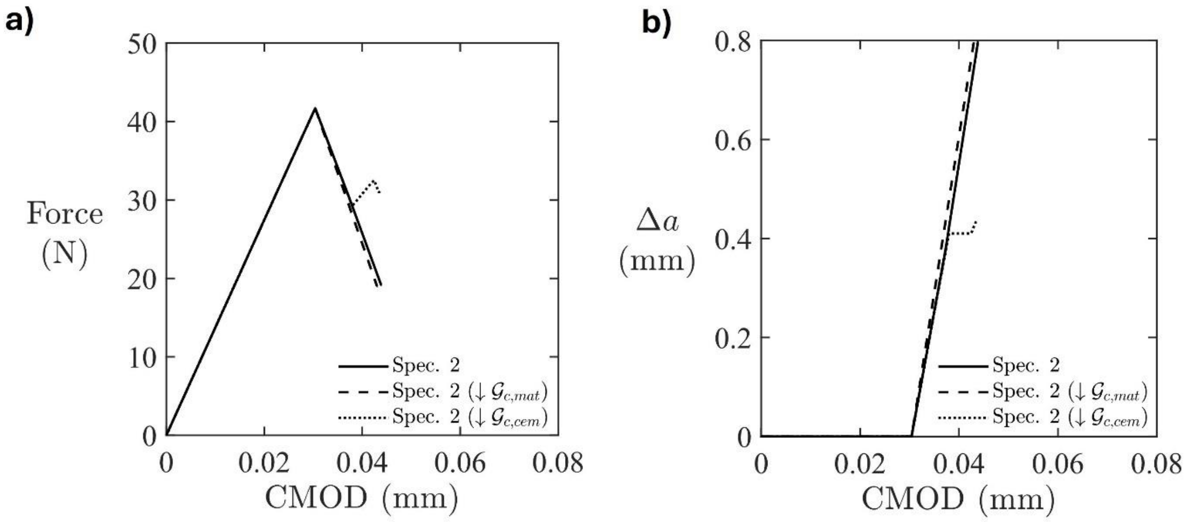

Fig. S7 (a) Force vs CMOD and (b) crack extension Δa vs CMOD curves for specimen 2: reference simulation ($\mathcal{G}_{c,mat} = 0.36$ N/mm and $\mathcal{G}_{c,cem} = 0.14$ N/mm), with reduced $\mathcal{G}_{c,mat}$ (↓ $\mathcal{G}_{c,mat}$ in the legend) in which $\mathcal{G}_{c,mat} = 0.35$ N/mm and $\mathcal{G}_{c,cem} = 0.14$ N/mm and with reduced $\mathcal{G}_{c,cem}$ (↓ $\mathcal{G}_{c,cem}$ in the legend) in which $\mathcal{G}_{c,mat} = 0.36$ N/mm and $\mathcal{G}_{c,cem} = 0.13$ N/mm. Other material parameters are given in Table 6.
